# Retinoic acid inhibits the angiogenesis of human embryonic stem cell-derived endothelial cells by activating FBP1-mediated gluconeogenesis

**DOI:** 10.1186/s13287-022-02908-x

**Published:** 2022-06-07

**Authors:** Zhuangzhuang Yang, Miao Yu, Xuechun Li, Yuanyuan Tu, Chunyan Wang, Wei Lei, Min Song, Yong Wang, Ying Huang, Fengyue Ding, Kaili Hao, Xinglong Han, Xuan Ni, Lina Qu, Zhenya Shen, Shijun Hu

**Affiliations:** 1 Department of Cardiovascular Surgery of the First Affiliated Hospital & Institute for Cardiovascular Science, Collaborative Innovation Center of Hematology, State Key Laboratory of Radiation Medicine and Protection, Suzhou Medical College, Soochow University, Suzhou, 215000 China; 2 State Key Laboratory of Space Medicine Fundamentals and Application, China Astronaut Research and Training Center, Beijing, 100094 China

**Keywords:** Endothelial cell, Angiogenesis, Retinoic acid, Glycometabolism

## Abstract

**Background:**

Endothelial cells are located in the inner lumen of blood and lymphatic vessels and exhibit the capacity to form new vessel branches from existing vessels through a process called angiogenesis. This process is energy intensive and tightly regulated. Glycolysis is the main energy source for angiogenesis. Retinoic acid (RA) is an active metabolite of vitamin A and exerts biological effects through its receptor retinoic acid receptor (RAR). In the clinic, RA is used to treat acne vulgaris and acute promyelocytic leukemia. Emerging evidence suggests that RA is involved in the formation of the vasculature; however, its effect on endothelial cell angiogenesis and metabolism is unclear.

**Methods:**

Our study was designed to clarify the abovementioned effect with human embryonic stem cell-derived endothelial cells (hESC-ECs) employed as a cell model.

**Results:**

We found that RA inhibits angiogenesis, as manifested by decreased proliferation, migration and sprouting activity. RNA sequencing revealed general suppression of glycometabolism in hESC-ECs in response to RA, consistent with the decreased glycolytic activity and glucose uptake. After screening glycometabolism-related genes, we found that fructose-1,6-bisphosphatase 1 (FBP1), a key rate-limiting enzyme in gluconeogenesis, was significantly upregulated after RA treatment. After silencing or pharmacological inhibition of FBP1 in hESC-ECs, the capacity for angiogenesis was enhanced, and the inhibitory effect of RA was reversed. ChIP-PCR demonstrated that FBP1 is a target gene of RAR. When hESC-ECs were treated with the RAR inhibitor BMS493, FBP1 expression was decreased and the effect of RA on angiogenesis was partially blocked.

**Conclusions:**

The inhibitory role of RA in glycometabolism and angiogenesis is RAR/FBP1 dependent, and FBP1 may be a novel therapeutic target for pathological angiogenesis.

**Supplementary Information:**

The online version contains supplementary material available at 10.1186/s13287-022-02908-x.

## Introduction

Cardiovascular diseases (CVDs), caused by heart and blood vessel disorders, have led to one-third of deaths worldwide in recent years [17]. Endothelial cells (ECs) lining the inner surface of both the heart and blood vessels are sentinels of cardiovascular health. Endothelial dysfunction is implicated in the pathogenesis of various CVDs including atherosclerosis, diabetes, and myocardial infarction [11, 12, 33]. In healthy adults, ECs are usually quiescent and exhibit limited angiogenesis, but can be activated by angiogenic signals, resulting in new blood vessel formation under pathologic conditions. However, excessive activation of quiescent endothelial cells promotes pathologic angiogenesis associated with cancer, visual blindness, and pulmonary arterial hypertension [14, 29, 32]. Although antiangiogenic therapy targeting vascular endothelial growth factor (VEGF) has become an attractive clinical strategy [28], its insufficient effectiveness and drug resistance limit its success [7]. Multiple combinations of agents that block different angiogenic signals may confer benefits, but there is still a concern that angiogenesis can recur through the compensatory effects of other angiogenic factors and promote more aggressive phenotypes [7, 23]. Therefore, more studies have been carried out to identify a more comprehensive antiangiogenic strategy and steer the vasculature toward a mature and normal phenotype [8, 13].

Angiogenesis is regulated by genetic signaling, including VEGF and Notch signaling [1, 21, 26]. As the main energy source in ECs, glycolysis is fundamental for EC proliferation, migration and sprouting [4, 9, 22]. Upon proangiogenic stimulation, ECs increase their glycolytic flux to meet the elevated needs of energy and biomass to support migration and proliferation, respectively. The most studied glycolytic enzyme in ECs is 6-phosphofructo-2-kinase/fructose-2,6-biphosphatase-3 (PFKFB3), which is upregulated by VEGF and converts fructose-6-phosphate (F6P) into fructose-2,6-bisphosphate (F2,6BP). Inactivation of PFKFB3 reduces EC glycolysis, decreases EC migration and proliferation, and impairs vessel sprouting in vivo and in vitro [4]. However, whether other key glycolytic enzymes can also regulate angiogenesis is still unclear.

Gluconeogenesis is a pathway with a result opposite that of glycolysis and includes some reversible reactions of glycolysis. Fructose-1,6-bisphosphatase 1 (FBP1), which catalyzes the conversion of fructose-1,6-bisphosphate (F1,6P_2_) to fructose 6-phosphate (F6P), is a rate-limiting enzyme in gluconeogenesis. It has been reported that loss of FBP1 is a critical carcinogenic event in basal-like breast cancer [6] and lung tumorigenesis [3]. Recent work suggested that FBP1 is a metabolic tumor suppressor in liver cancer and that loss of FBP1 disrupts liver metabolic homeostasis and promotes tumor progression [18]. Collectively, these findings provided genetic evidence for FBP1 as a suppressor in a variety of cancers and suggested a genetic therapy for FBP1-induced improvement of gluconeogenesis. However, whether FBP1-driven gluconeogenesis affects angiogenesis remains to be determined.

Retinoic acid (RA) is an active metabolite of vitamin A that has been extensively investigated [24, 25] and is widely known for its anti-inflammatory effects [10, 16]. There are a limited number of reports focusing on its angiogenetic role [15, 30]; however, there is no report investigating how RA regulates glycometabolism in ECs. A previous study found that the expression of a retinoic acid signature reduced the capacity of circulating CD34^+^ cells from coronary artery disease patients to migrate to ischemic tissues [31]. Thus, we hypothesized that there are consequent associations between RA and angiogenesis; moreover, the mechanism of RA in vascular formation warrants elucidation.

Human embryonic stem cell-derived endothelial cells (hESC-ECs) have become a useful tool to study physiological and pathological vascular development [2, 34]. In this study, we investigated the role of RA in hESC-ECs and found that RA reduced EC angiogenesis, as reflected by the reduced sprouting, migration, and proliferation capacities, through facilitating FBP1-driven gluconeogenesis and reducing glycolytic flux.

## Materials and methods

### Cell culture and differentiation

H1 human embryonic stem cells (WiCell Research Institute, USA) were cultured in PSCeasy medium (Cellapy, China) in Matrigel-coated dishes (Corning, USA) and passaged with 0.5 mM EDTA at 70–80% confluence. At ~ 90% confluence, H1 cells were moved into CDM3 medium with different supplements to induce directional differentiation. CDM3 medium consists of RPMI 1640 medium (Thermo Fisher, USA), recombinant human albumin (Sigma-Aldrich, USA) and L-ascorbic acid 2-phosphate (Sigma-Aldrich, USA). During days 0–2, the medium was supplemented with 6 μM CHIR99021 (Sigma-Aldrich, USA). On day 2, 50 ng/mL bFGF (Novoprotein, USA) was added. After that, 50 ng/mL VEGF (Novoprotein, USA), 25 ng/mL BMP4 (PeproTech, USA) and 25 ng/mL bFGF (Novoprotein, USA) were used for the next 3 days. The medium was changed daily. On day 6, CD34^+^ cells were enriched using anti-CD34 antibody-conjugated magnetic beads (Miltenyi, USA) according to the manufacturer’s instructions. The sorted cells were defined as human embryonic stem cell-derived endothelial cells (hESC-ECs).

### Flow cytometry

Cells were digested with 0.1% trypsin and filtered through a 30 μm nylon mesh (Fisher Scientific, USA) to remove cell clumps. Then, cells were washed once with PBS and resuspended in 90 μL of 3% BSA containing APC-conjugated anti-CD34 and PE-conjugated anti-CD144 antibodies according to the manufacturer’s instructions. After a 30-min incubation, cells were analyzed using a Guava EasyCyte™ 8 flow cytometer (EMD Millipore, Germany). The antibodies used for flow cytometry are listed in Additional file 1: Table S1.

### Immunofluorescence staining

Cultured cells were fixed with 4% paraformaldehyde for 10 min, blocked with blocking buffer (3% FBS, 1% BSA, 0.5% Tween-20 and 0.5% Triton X-100 in PBS) for 1 h at room temperature and then incubated with primary antibodies overnight at 4 °C. After three 5-min washes in PBST, cells were incubated with the corresponding secondary antibodies in 3% BSA for 2 h at room temperature in the dark. Finally, after three 5-min washes in PBST, cells were counterstained with Hoechst 33342 for 10 min, imaged with a Zeiss LSM880 confocal laser scanning microscope (Carl Zeiss, Germany) and further analyzed using ImageJ software. The antibodies used for immunofluorescence are listed in Additional file 1: Table S1.

### Dil-ac-LDL uptake assay

hESC-ECs were incubated with Dil fluorescent dye-labeled acetylated low-density lipoprotein (Dil-Ac-LDL) (Biomedical Technologies, USA) at 10 μg/mL in ECM (ScienCell, USA) for 4 h at 37 °C. After washing three times with PBS, cells were subjected to immunofluorescence staining, and images were acquired with a Zeiss LSM880 confocal laser scanning microscope (Carl Zeiss, Germany).

### Spheroid-based sprouting assay

The spheroid-based sprouting assay was conducted as previously described with further optimization [35]. Briefly, hESC-ECs were seeded at a density of 400 cells per well into an ultra-low attachment 96-well plate (Corning, USA) and cultured in ECM containing 20% methylcellulose (Sigma-Aldrich, USA) for spheroid formation. After at least 24 h, the formed spheroids were embedded in a collagen matrix and cultured in ECM for another 24 h. Images of 10 randomly selected spheroids were acquired under an inverted microscope. The sprout number and sprout length were analyzed with ImageJ software.

### Scratch assay

A scratch assay was performed to analyze cell migration in vitro as previously described with further optimization [19]. hESC-ECs were seeded at a density of 5 × 10^3^ cells per well in a 48-well plate to form a confluent monolayer. The next day, a scratch wound was created by scraping the cell monolayer in a straight line with a p200 pipette tip. After washing with D-PBS, hESC-ECs were treated as indicated. At the end of the experiment, images were acquired under an inverted microscope. The relative scratch area (with respect to the initial scratch area) and migration ratio (migration area/initial scratch area) were measured with ImageJ software.

### Cell cycle analysis

EdU incorporation (DNA synthesis) and PI staining (DNA content) were jointly used to analyze the cell cycle. Briefly, viable hESC-ECs were first incubated with EdU in an incubator for 4 h. After fixation and permeabilization, EdU-labeled cells were incubated sequentially with Click Reaction cocktail provided in the BeyoClick™ EdU Cell Proliferation Kit with Alexa Fluor 647 (Beyotime Biotechnology, China) and PI. Alexa Fluor 647 and PI fluorescence were detected using a Guava easyCyte™ 8 flow cytometer (EMD Millipore, Germany).

### RNA sequencing

After RNA extraction from hESC-ECs treated with or without RA, 3 µg of total RNA per sample was used for library preparation, and RNA sequencing was performed on the BGISEQ-500 platform. The sequencing data were filtered with SOAPnuke (v1.5.2) by removing reads (1) containing sequencing adapters, (2) whose low-quality base ratio (base quality less than or equal to 5) was greater than 20%, and (3) whose unknown base ratio was greater than 5%; then, clean reads were obtained and stored in FASTQ format. The clean reads were mapped to the reference genome using HISAT2 (v2.0.4). Bowtie2 (v2.2.5) was applied to align the clean reads to the reference coding gene set, and the expression levels of the genes were then calculated with RSEM (v1.2.12). The heatmap was generated with heatmap (v1.0.8) according to the gene expression levels in different samples. In addition, differential expression analysis was performed using DESeq2 (v1.4.5) with a *Q* value ≤ 0.05 considered to indicate significantly differential expression. To gain insight into changes in phenotype, GO enrichment analysis of annotated differentially expressed genes were performed with Phyper based on the hypergeometric distribution. The significance levels for terms and pathways were determined by calculating the corrected *Q* values with a rigorous threshold (*Q* value ≤ 0.05) from the Bonferroni-corrected *P* values.

### Glycolytic flux assay

After the indicated treatment, hESC-ECs were seeded at a density of 6 × 10^4^ cells per well in sextuplicate into a gelatin-coated Seahorse XF24 culture plate. The extracellular acidification rate (ECAR) was measured the next day with a Seahorse XF24 extracellular flux analyzer (Seahorse Bioscience, USA) to indicate glycolytic flux as previously described [20]. Before measurement, cells were incubated in unbuffered assay medium (Sigma, USA) for 1 h in a non-CO_2_ incubator at 37 °C. ECAR was measured for five cycles. Each cycle consisted of a 3-min medium mixing time, a 2-min waiting time and a 2-min measurement time. Measurements were taken at four successive timepoints: before the addition of glucose (10 mM), before the addition of oligomycin (3 µM) and before and after the addition of the glycolysis inhibitor 2-DG (100 mM). All compounds were prepared in assay medium and adjusted to pH 7.4. ECAR values were normalized to the protein content.

### Glucose uptake assay

Glucose uptake was measured using the fluorescence-labeled deoxyglucose analog 2-(*N*-(7-nitrobenz-2-oxa-1,3-diazol-4-yl) amino)-2-deoxyglucose (2-NBDG, Apex Bio, USA). After the indicated treatment, hESC-ECs were incubated with 2-NBDG for 1 h at 37 °C, and the fluorescence intensity was measured using the Guava EasyCyte™ 8 flow cytometer (EMD Millipore, Germany).

### RNA isolation and real-time PCR

After the indicated treatment, total RNA was extracted using TRI Reagent (Sigma-Aldrich, USA) according to the manufacturer’s instructions. Equal amounts of total RNA from each sample were used to synthesize first-strand cDNA with a reverse transcription kit (Takara, Japan) prior to real-time PCR using SYBR Green PCR Master Mix (Thermo Fisher, USA). *18S rRNA* was used to normalize gene expression data. Relative expression levels were calculated using the 2^−ΔΔCT^ method. The primer sequences used in this assay are listed in Additional file 1: Table S2.

### ChIP-PCR

The ChIP assay was performed using an EZ-ChIP kit (Millipore, USA) according to the manufacturer’s protocol as routinely performed [20]. Antibodies against RARα (Abcam, USA) were used to immunoprecipitate sheared, crosslinked chromatin. Based on the JASPAR database, three potential retinoic acid response elements (RAREs) were predicted in the promoter region of human *FBP1*. The three RAREs were named RARE1 (bp − 1588 to bp − 1571: 5′-AGGGTTATCTGGGGGCGA-3′), RARE2 (bp − 1004 to bp − 987: 5′-TGACTCTGAGATGCCCTC-3′) and RARE3 (bp − 164 to bp − 151: 5′-TGACTCTGAGATGCCCTC-3′). The primers used for ChIP-PCR are listed in Additional file 1: Table S3, with human *GAPDH* as the internal reference.

### Western blot analysis

After the indicated treatment, total protein extraction was conducted using RIPA lysis buffer prior to protein quantification using a BCA assay. After that, equal amounts of total protein for each sample were separated by SDS-PAGE and transferred onto a PVDF membrane. The membrane was probed with an antibody against FBP1 and visualized with a Phototope™-HRP Western Blot Detection System (Cell Signaling Technology, USA), with β-actin used as the internal reference. The antibodies used for western blot are listed in Additional file 1: Table S1.

### Gene knockdown assay

Small interfering RNAs (siRNAs) directed against human *FBP1* were synthesized and transfected into hESC-ECs to inhibit the expression of FBP1, with a scrambled siRNA used as a control. The sequences of the siRNAs targeting human *FBP1* are listed in Additional file 1: Table S4.

### In vivo Matrigel plug assay

Male SCID mice aged 8 weeks were used to conduct the in vivo Matrigel plug assay with the approval of Ethics Committee Soochow University (SUDA20220120A01). 12 mice were divided randomly into two groups (*n* = 6 for each group). 0.5 × 10^7^ cells treated with or without retinoic acid resuspended in 200 μL Matrigel were injected subcutaneously into the flank region. All mice were euthanized at 10 days after injection. Matrigel plugs were photographed and removed for the following H&E staining and immunostaining for CD31 to evaluate angiogenesis.

### Statistical analysis

All experimental assays were performed in at least triplicate, and the values are presented as the means ± SEMs. Comparisons between two groups were analyzed using Student’s *t* test. Comparisons among multiple groups were analyzed using one-way analysis of variance (ANOVA) followed by the Bonferroni post hoc test. *P* values less than 0.05 were considered statistically significant.

## Results

### RA inhibited angiogenesis

**Fig. 1:**
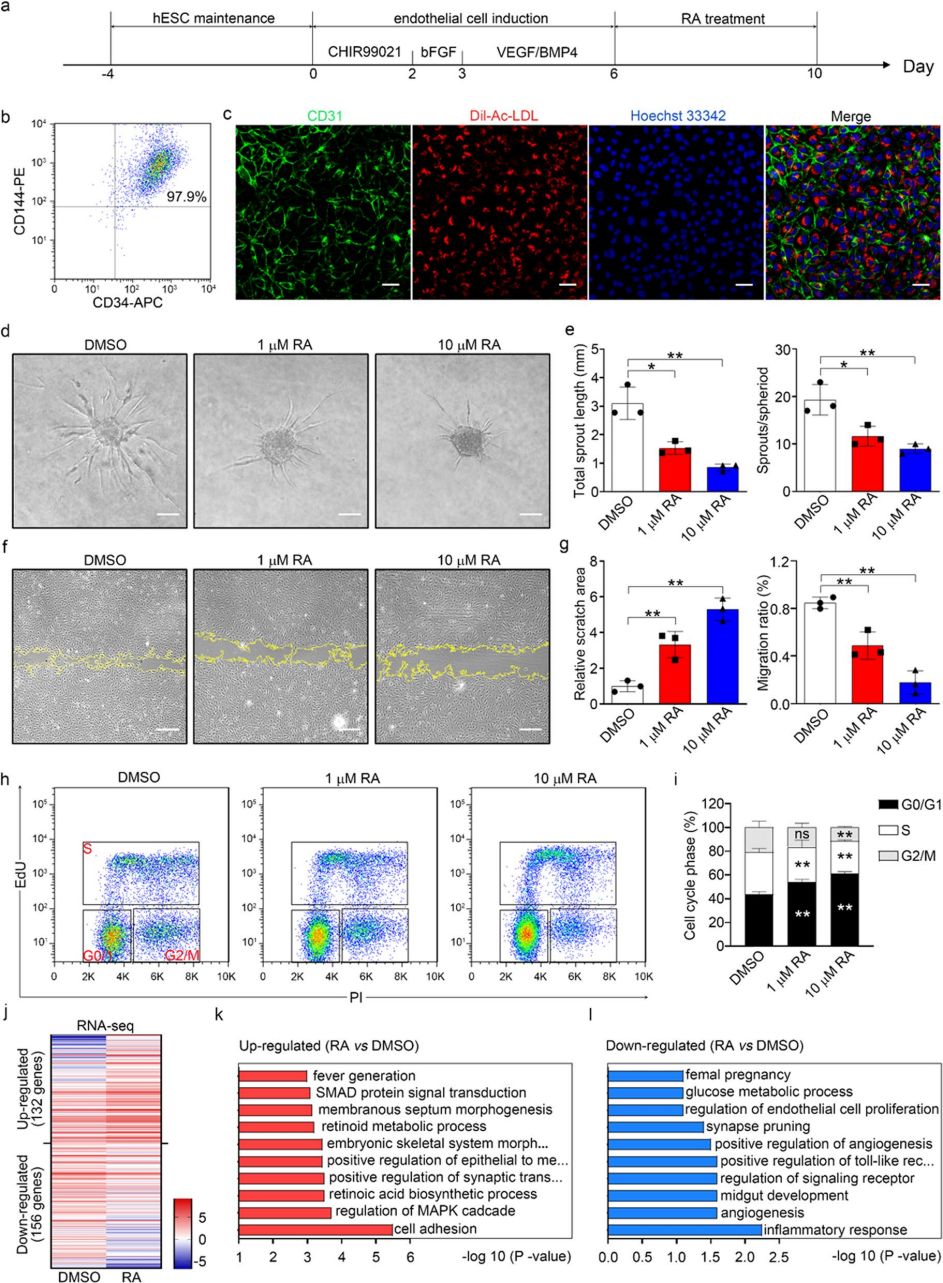
RA inhibited angiogenesis. **a** Schematic diagram of the process for differentiating human embryonic stem cells into endothelial cells. **b** Representative flow cytometric plots of the expression of the endothelial markers CD144 and CD34 after sorting with an anti-CD34 antibody. **c** Representative photographs of immunocytochemistry of human CD31 (green) and Dil-ac-LDL (red) staining. Nuclei were stained with Hoechst 33342. Scale bar, 50 µm. **d** Representative photographs of endothelial spheroids. Scale bar, 100 μm. **e** Statistical analysis of the total sprout length and sprout/spheroid ratio. **f** Representative photographs of wound healing. Scale bar, 100 µm. **g** Statistical analysis of the scratch area and migration rate. **h** Cell cycle analysis. **i** Statistical analysis of different cell cycle phases. **j** Correlation heatmap of the transcript levels of 288 significantly differentially expressed genes in RA-treated ECs versus control ECs. Color scale: red, high correlation; blue, low correlation. **k** GO biological processes enriched with upregulated genes. Significance is shown as—log10 Bonferroni-corrected *P* value after multiple hypothesis correction. **l** GO biological processes enriched with downregulated genes. Significance is shown as the—log10 Bonferroni-corrected *P* value after multiple hypothesis correction. All data are presented as mean ± SEM; One-way ANOVA; *p < 0.05, **p < 0.01, and ns, not significant

Stepwise differentiation of human embryonic stem cells into endothelial cells (hESC-ECs) was driven by sequential treatment with CHIR99021, bFGF, VEGF and BMP4 as indicated in Fig. 1a. As differentiation progressed, the expression levels of the endothelial markers *KDR, CD34, CD31* and *CD144* increased, while stemness was gradually lost, as indicated by the decreased levels of *NANOG* and *OCT4* (Additional file 1: Fig. S1a–f). CD34 is a transmembrane glycoprotein and is expressed on endothelial cells. After 6 days of differentiation, hESC-ECs were isolated using anti-CD34 antibody-conjugated magnetic beads. Flow cytometric analysis demonstrated that approximately 98% of the purified cells were CD144^+^/CD34^+^ (Fig. 1b). hESC-ECs were maintained in ECM supplemented with VEGF, bFGF and a low concentration of serum. The purified ECs expressed the endothelial lineage marker CD31, as revealed by immunostaining, and exhibited the capacity for Dil-Ac-LDL uptake (Fig. 1c) and tube formation (Additional file 1: Fig. S2a). Moreover, hESC-ECs exhibited an mRNA expression profile similar to that of HUVECs (Additional file 1: Fig. S2b–d). hESC-ECs were differentiated from multipotential stem cells. The differentiation method is standard and repeatable. They are relatively easy to remain and renew. They can be seen as a good cell resource to transform in clinic. Collectively, these results indicated that hESC-ECs are a reliable cell model for conducting the following experiments.

To comprehensively study the effect of RA on angiogenesis, spheroid-based sprouting assay, tube formation assay, scratch wound assay, cell proliferation assay and cell cycle analysis were conducted. As indicated in Fig. 1d–e, sprouting of EC spheroids was blocked after RA treatment, as evidenced by the decreased sprout number and sprout length. Moreover, RA impaired tube formation, as shown by the decreased tube length and number of branch points (Additional file 1: Fig. S3a–c). As EC migration is essential for angiogenesis, a scratch wound assay was used to evaluate migration. We found that RA significantly repressed EC migration, as shown by the decreased migration ratio and increased scratch area (Fig. 1f–g). Similar inhibitory effects were observed in HUVECs after RA treatment as shown in Additional file 1: Fig. S3g–h. It indicates that the inhibitory role of RA for angiogenesis is universal or at least not cell-type-specific. Then, the proliferation ability of hESC-ECs was evaluated. We found that RA treatment decreased EdU incorporation, formazan dye formation in the CCK8 assay and the cell count (Additional file 1: Fig. S3d–f), indicating decreased proliferation activity. *P16*, a cyclin-dependent kinase inhibitor, has been reported to prevent S-phase entry. We found that RA significantly upregulated *P16* mRNA expression (Additional file 1: Fig. S3i), implying that RA might induce cell cycle arrest in ECs. However, we did not find significant difference between the cells with or without RA treatment in the degree of senescence as detected by β-gal staining (Additional file 1: Fig. S3j). These results prompted us to analyze the cell cycle in RA-treated ECs. We analyzed the cell cycle by flow cytometry using EdU (which is incorporated into newly synthesized DNA) and PI (which labels DNA). We found that RA increased the percentage of G0/G1-phase cells and decreased that of S-phase cells (Fig. 1h–i), which was in line with the abovementioned increase in the *P16* level. Moreover, the inhibitory role of RA in EC proliferation/migration/sprouting was dose-dependent.

### RA reduced glycolytic flux

To gain unbiased insight into the potential mechanisms by which RA inhibits angiogenesis, we performed RNA sequencing on hESC-ECs treated with RA or not. Through bioinformatic analysis, we identified a total of 288 differentially expressed genes (*p* < 0.05, fold change ≥ 2), namely 132 upregulated and 156 downregulated genes (Fig. 1j; Additional file 1: Fig. S4). The top GO terms enriched with upregulated genes were mainly terms associated with RA biosynthesis, metabolic processes, and non-endothelium-related pathways (Fig. 1k). The top GO terms enriched with downregulated genes were associated with angiogenesis, positive regulation of angiogenesis and regulation of endothelial cell proliferation (Fig. 1l), further verifying the above phenotype in RA-treated ECs.

**Fig. 2:**
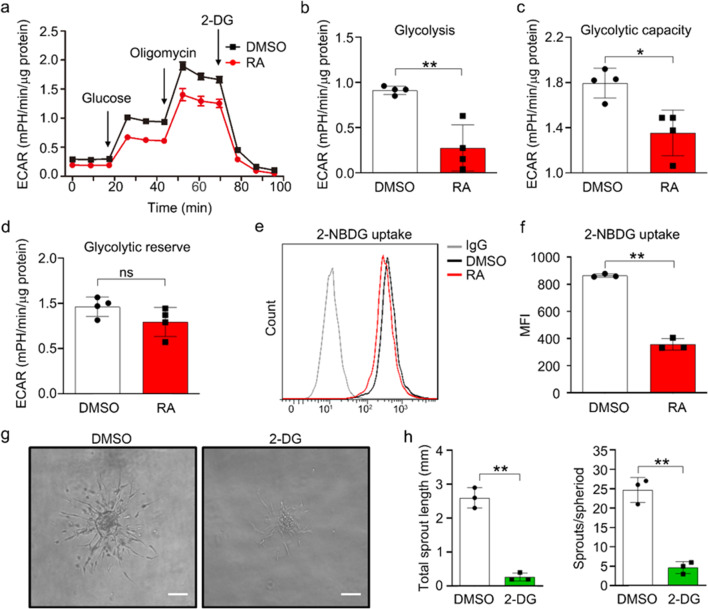
RA reduced endothelial glycolysis. **a** Extracellular acidification rate (ECAR) profile in DMSO and RA-treated hESC-ECs. The vertical lines indicate the times of addition of glucose (10 mmol/L), oligomycin (3 μmol/L), and 2-deoxy-D glucose (100 mmol/L). Quantification of glycolysis (**b**), glycolytic capacity (**c**), and glycolytic reserve (**d**) for the cells described in panel (**a**). (**e**) Representative flow cytometric overlay and quantification of 2-NBDG uptake in RA-treated hESC-ECs compared with control hESC-ECs, as measured by flow cytometric analysis of 2-NBDG fluorescence. **f** Statistical analysis of the data in panel **e** showing significantly decreased 2-NBDG uptake. **g** Representative photographs of endothelial spheroids showing reduced sprouting in 2-DG-treated hESC-ECs. Scale bar, 100 μm. **h** Statistical analysis of the data in panel **g** showing a significantly decreased total sprout length and sprout/spheroid ratio. All data are presented as mean ± SEM; Student’s t test; *p < 0.05, **p < 0.01, and ns, not significant

Considering that angiogenesis is an energy-intensive process and that endothelial cells rely on glycolysis to generate most (~ 85%) of their ATP [4], the downregulated glucose metabolic process attracted our attention and encouraged us to test glycolytic flux with a Seahorse XF24 extracellular flux analyzer. ECAR is an indicator of glycolysis. As shown in Fig. 2a, the ECAR in the RA-treated group was still lower than that in the DMSO group (Fig. 2a). After quantitative analysis, we found that RA significantly inhibited glycolysis and the glycolytic capacity, leaving the glycolytic reserve unchanged (Fig. 2b–d). Next, we examined the effect of RA on glucose uptake. The fluorescence-labeled 2-deoxyglucose analog 2-NBDG was used to trace glucose uptake. The results of flow cytometry indicated that RA treatment suppressed glucose uptake (Fig. 2e–f). Since RA reduced glycolytic flux, we sought to determine whether glycolysis blockade impairs endothelial sprouting. 2-DG is one of the most relevant glycolysis inhibitors. As indicated in Fig. 2g–h, 2-DG treatment inhibited EC spheroid sprouting, as evidenced by the decreased sprout number and length. Collectively, these data revealed that RA treatment reduces glycolytic flux in hESC-ECs and that this effect is related to angiogenesis inhibition.

### RA increased gluconeogenesis by upregulating FBP1

**Fig. 3:**
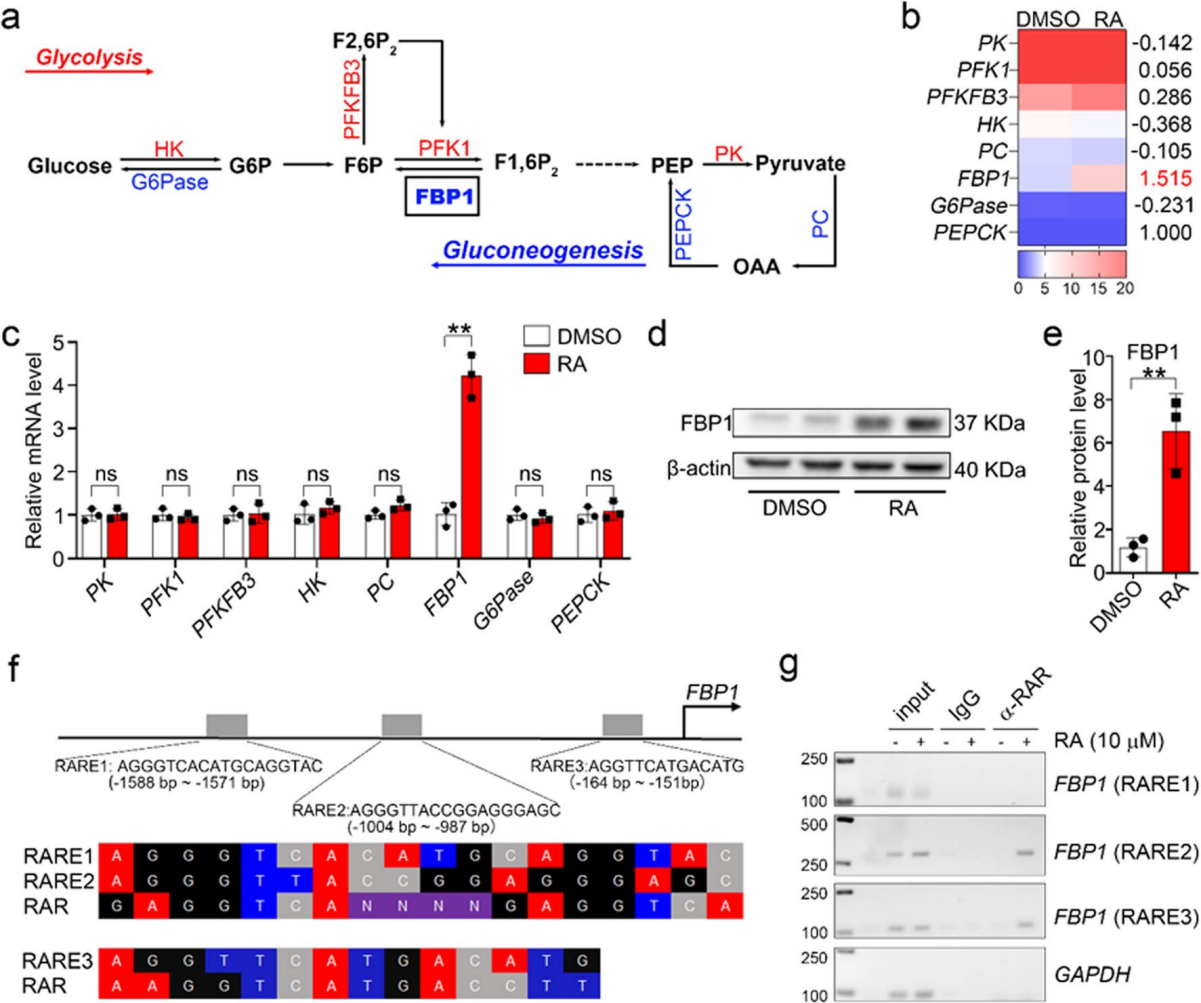
RA increased gluconeogenesis by upregulating FBP1. **a** Pathway map of glycolytic and gluconeogenic metabolism. (G6P: glucose-6-phosphate; F6P: fructose-6-phosphate; F1,6P_2_: fructose-1,6-bisphosphate; F2,6P_2_: fructose-2,6-bisphosphate; PEP: phosphoenolpyruvate; OAA: oxaloacetate; HK: hexokinase; G6Pase: Glucose-6-phosphatase; PFKFB3: 6-phosphofructo-2-kinase/fructose-2,6-bisphosphatase 3; PFK1: phosphofructokinase-1; FBP1: fructose-1,6-biphosphatase1; PK: pyruvate kinase; PC: pyruvate carboxylase; PEPCK: phosphoenolpyruvate carboxykinase. **b** FPKM quantification of genes encoded for the 8 rate-limiting enzymes defined in panel (**a**). **c** The qPCR analysis of the mRNA expression of genes encoded for the 8 rate-limiting genes. **d** Representative western blot of FBP1. **e** The quantitative analysis of FBP1 expression. **f** Sequence alignment of the three predicted RAREs (p-RAREs) to the standard RARE (s-RARE) and their location in the *FBP1* promoter region. **g** ChIP-PCR revealed the direct binding of RARα to RAREs in the *FBP1* promoter in hESC-ECs after RA treatment**.** The three RAREs in the *FBP1* promoter were named RARE1 (bp − 1588 to bp − 1571: 5′-AGGGTTATCTGGGGGCGA-3′), RARE2 (bp − 1004 to bp − 987: 5′-TGACTCTGAGATGCCCTC-3′) and RARE3 (bp − 164 to bp − 151: 5′-TGACTCTGAGATGCCCTC-3′). All data are presented as mean ± SEM; Student’s t test; **p < 0.01, and ns, not significant

Considering that the glucose metabolic process was downregulated after treatment, we assessed the performance of 8 rate-limiting enzymes in glycolysis (in red) and gluconeogenesis (in blue), as shown in Fig. 3a. Gluconeogenesis is considered a process with a result opposite that of glycolysis. We found that fructose 1,6-bisphosphatase 1 (FBP1) in gluconeogenesis was the most significantly upregulated candidate, and no significant changes were observed for the other 7 rate-limiting enzymes (Fig. 3b–c). FBP1 catalyzes the conversion of fructose-1,6-bisphosphate (F1,6P_2_) to fructose 6-phosphate (F6P) and negatively regulates glycolysis. Correspondingly, RA treatment stimulated FBP1 protein expression (Fig. 3d). Taken together, these results indicated that RA stimulates FBP1 and eventually leads to a reduction in glycolytic flux, energy supply and angiogenesis.

RA exerts its biological effects by binding to retinoic acid receptors (RARs) and retinoid X receptors (RXRs). RAR-RXR heterodimers bind to specific DNA regions, termed retinoic acid response elements (RAREs), in the promoter regions of target genes, leading to transcriptional control. Three potential RAREs in the *FBP1* promoter region were predicted by the JASPAR database (Fig. 3d). The three RAREs in the *FBP1* promoter were named RARE1 (bp − 1588 to bp − 1571: 5′-AGGGTTATCTGGGGGCGA-3′), RARE2 (bp − 1004 to bp − 987: 5′-TGACTCTGAGATGCCCTC-3′) and RARE3 (bp − 164 to bp − 151: 5′-TGACTCTGAGATGCCCTC-3′). ChIP-PCR revealed the sites of RAR-FBP1 interaction with RARE2/3 as the genomic binding sites (Fig. 3f). Collectively, these results demonstrated that FBP1 is a novel target gene of RA.

### Inhibition of FBP1 stimulated angiogenesis

**Fig. 4:**
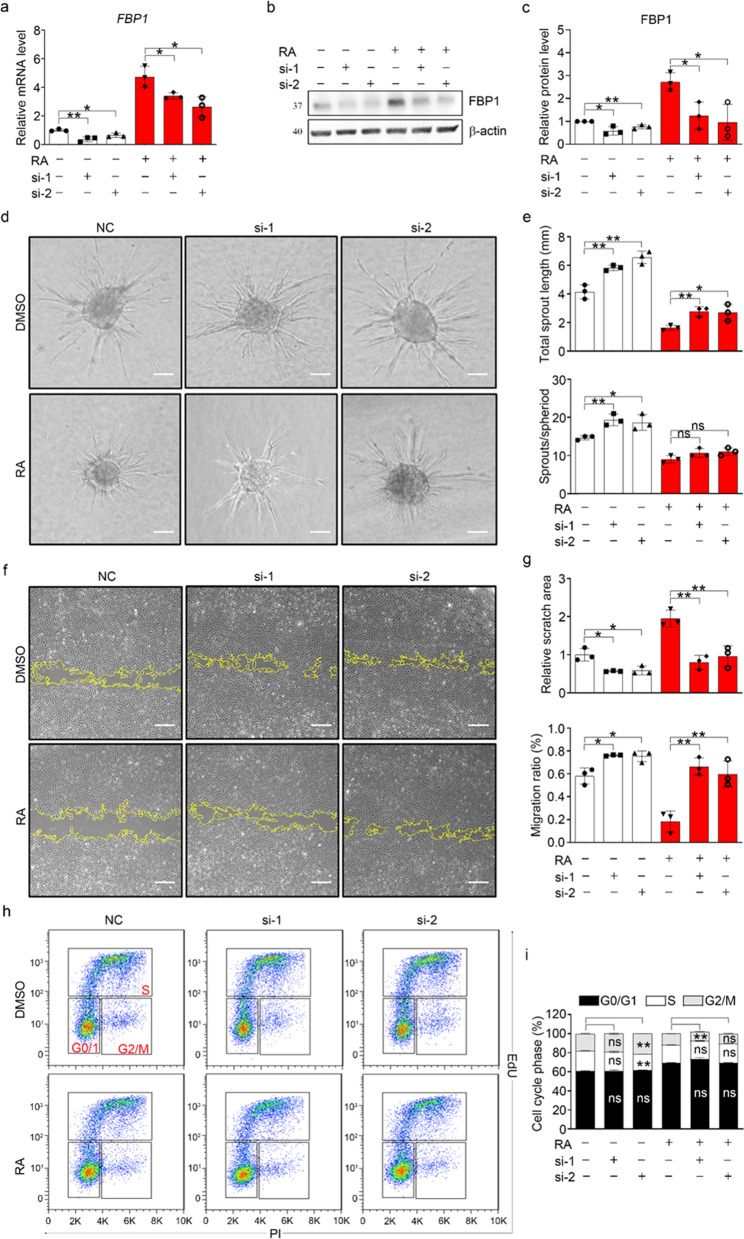
Inhibition of FBP1 stimulated angiogenesis. **a** The qPCR analysis of FBP1 mRNA expression. **b** Representative western blot of FBP1. **c** Quantification of the FBP1 protein level in panel (**b**). **d** Representative bright-field images of EC spheroids. Scale bar, 100 μm. **e** Statistical analysis of the data in panel (**d)** for the total sprout length and sprout/spheroid ratio. **f** Representative bright-field images of wound healing. Scale bar, 100 μm. **g** Statistical analysis of the data in panel **f** for the scratch area and migration rate. **h** Cell cycle analysis of endothelial cells. **i** Statistical analysis of the data in panel (**h**). All data are presented as mean ± SEM; One-way ANOVA; *p < 0.05, **p < 0.01, and ns, not significant

To further confirm the role of FBP1 in mediating the inhibitory effect of RA on angiogenesis in hESC-ECs, we performed selective knockdown of *FBP1* using two small interfering RNAs (si-1 and si-2). FBP1 mRNA and protein expression were found to be significantly downregulated after si-FBP1 transfection in both the control and RA-treated groups (Fig. 4a–c). Functionally, inhibition of FBP1 significantly enhanced the sprouting, migration, and proliferation of hESC-ECs (Fig. 4d–i), implying activation of angiogenesis. Moreover, FBP1 inhibition partially counteracted the inhibitory effect of RA on angiogenesis. Pharmacological blockade of FBP1 with its inhibitor (20 µM, Cayman Chemical, USA) had an effect similar to that of si-FBP1 (Additional file 1: Fig. S5d–e). Thus, we concluded that inhibition of FBP1 stimulates angiogenesis and that RA exerts its inhibitory effect on angiogenesis in an FBP-1-dependent manner.

### RAR inhibition restored angiogenesis.

**Fig. 5:**
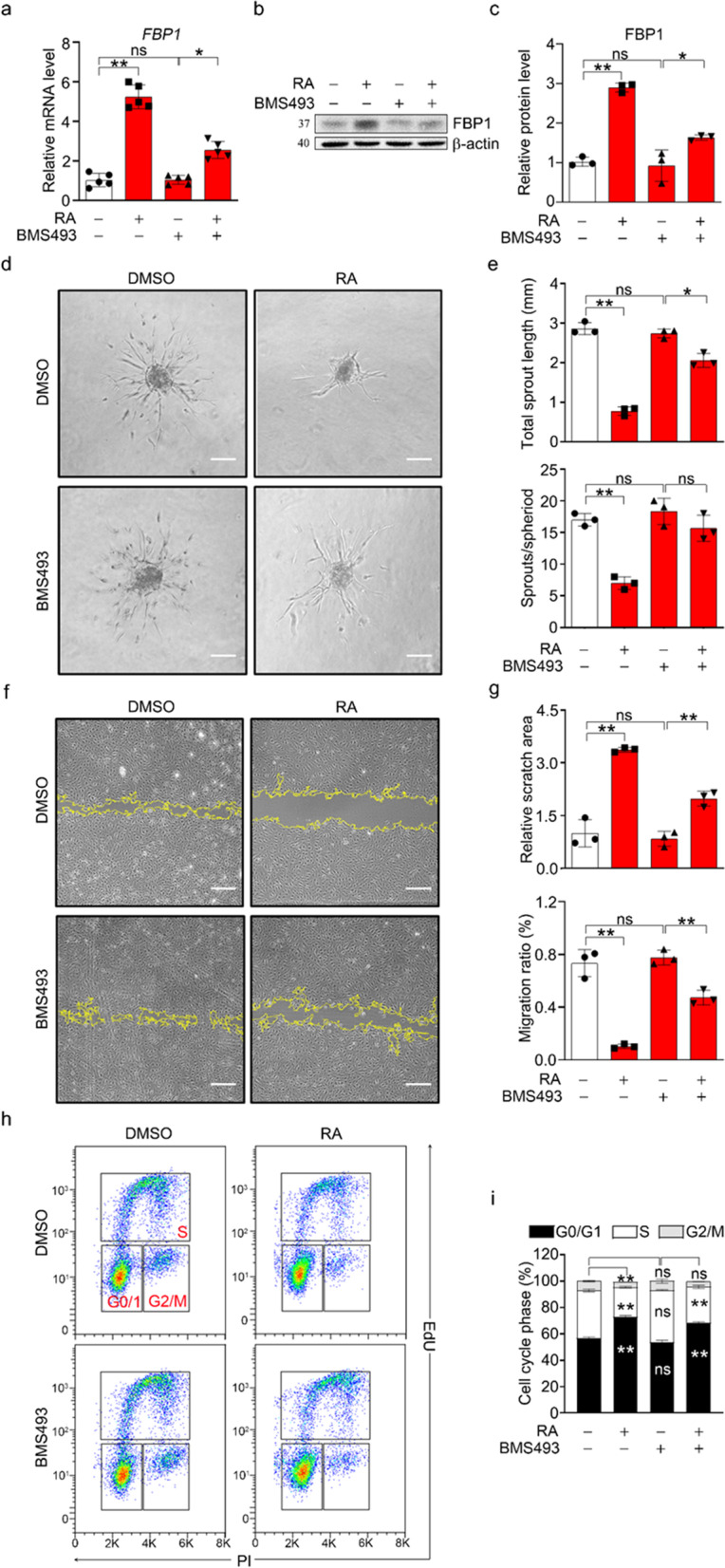
RAR inhibition restored angiogenesis. **a** The qPCR analysis of FBP1 mRNA expression. **b** Representative western blot of FBP1. **c** Quantification of the FBP1 protein level in panel (**b**). **d** Representative bright-field images of EC spheroids. Scale bar, 100 μm. **e** Statistical analysis of the data in panel **d** for the total sprout length and sprout/spheroid ratio. **f** Representative bright-field images of wound healing. Scale bar, 100 μm. **g** Statistical analysis of the data in panel **f** for the scratch area and migration rate. **h** Cell cycle analysis of endothelial cells. **i** Statistical analysis of the data in panel (**h**). All data are presented as mean ± SEM; One-way ANOVA; *p < 0.05, **p < 0.01, and ns, not significant

Since RA exerts its biological effects by binding to RARs, we sought to determine whether the inhibitory role of RA on angiogenesis is RAR dependent. We treated hESC-ECs with the small molecule compound BMS493, a pan-RAR inhibitor. In cells cotreated with 1 µM BMS493 (Tocris Bioscience, USA), RA lost its ability to induce FBP1 expression (Fig. 5a–c). As a result, BMS493 significantly restored the sprouting (Fig. 5d–e), tube formation (Additional file 1: Fig. S6a–c), migration (Fig. 5f–g) and proliferation (Fig. 5h–i; Additional file 1: Fig. S6d–e) activities inhibited by RA. BMS493 alone did not affect FBP1 expression and had no effect on angiogenesis. Taken together, our data revealed an inhibitory effect of the RA-RAR-FBP1 cascade on angiogenesis.

### RA inhibited angiogenesis in vivo

**Fig. 6:**
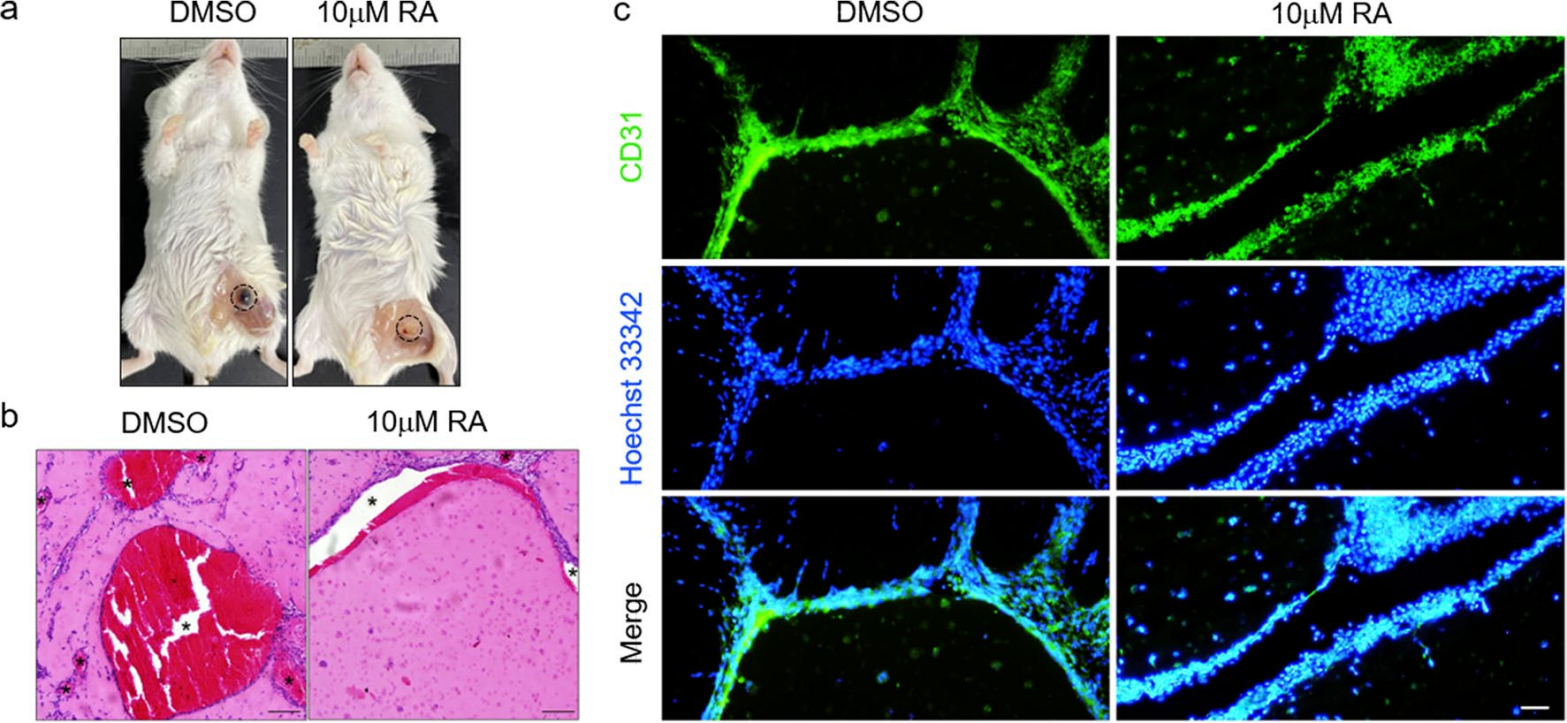
RA inhibits angiogenesis in vivo. **a** Representative images of Matrigel plug as highlighted in circle. The intensity of the red color and the level of transparency are parameters for qualitative evaluation. **b** Representative images of Matrigel plug paraffin section. Cells and Nuclei are stained with H&E. Matrigel is stained for pale background color. Vessel lumens are characterized by the absence of staining or filled with red blood and marked with asterisks. Scale bar, 100 μm. **c** Representative images of Matrigel plug paraffin section. Endothelial cells are stained for CD31 (green) and nuclei are stained with Hoechst 33,342 (blue). Scale bar, 50 μm

To detect whether the inhibitory role of RA for angiogenesis still holds in vivo, the Matrigel plug assay was conducted in male SCID mice at age of 8 week. 0.5 × 10^7^ cells treated with or without RA resuspended with 200 μL Matrigel were injected subcutaneously into the flank region. The Matrigel plug formed rapidly and the injected mice were euthanized at 10 days after injection. As the general view shown in Fig. 6a, the intensity of red color of the plug in RA-treated group was much more lower than that in control group, while the level of transparency was significantly higher. These two parameters indicated the declined angiogenesis level. The representative images of H&E staining and immunostaining for CD31 revealed some more detailed information as shown in Fig. 6b and c. Much more vessel lumens and branches were formed in control group and they are more mature to some extent compared to RA group. As for endothelial staining, antibody against CD31 can only react with antigen from human to exclude the potential disturbance from mice. The abovementioned results demonstrated the inhibitory role of RA for angiogenesis in vivo.

## Discussion

In this study, we investigated the role of RA in angiogenesis in hESC-ECs from a metabolic perspective. The results showed that RA reduced hESC-EC sprouting, migration and proliferation. These effects occurred partially due to RA-mediated facilitation of FBP1-driven gluconeogenesis and reduction of glycolytic flux. Moreover, our data indicated that RA upregulated FBP1 expression by directly binding to the FBP1 promoter via RAREs, and we suggested that FBP1 was a novel target gene of RA. These effects of RA on gluconeogenic metabolism and sprouting in hESC-ECs could offer a novel target for antiangiogenic therapy in angiogenesis-related pathological processes.

Retinoic acid is an active metabolite of vitamin A and directly regulates mammalian vessel formation during embryogenesis and adulthood [25]. A previous study showed that RA signaling regulates vasculogenesis during embryogenesis in mice deficient in retinaldehyde dehydrogenase 2 (*Raldh2*^–/–^), the enzyme required to produce active RA [15]. Moreover, RA was shown to inhibit microvasculature formation in chickens by downregulating Tie2 signaling [30]. Previous data indicated that RA altered the gene expression profile of CD34^+^ cells in coronary artery disease patients in a manner related to activation of differentiation via a retinoic acid-induced differentiation program, suggesting that circulating CD34^+^ cells in coronary artery disease patients are programmed by RA, leading to a reduced capacity to migrate to ischemic tissues, which in turn may be a reason for the reduced angiogenesis [31]. Using human embryonic stem cell-derived endothelial cells, we found similar results: RA reduced proliferation by increasing the *P16* level, which caused cell cycle arrest and quiescence. RA also inhibited the migration and sprouting of hESC-ECs, and we attributed these effects to the RA-induced reduction in glycolysis. Using ChIP-PCR, we identified that RA directly upregulates the expression of FBP1, a rate-limiting gene in gluconeogenesis; we proved that upregulated FBP1 expression downregulates glycolysis in hESC-ECs and causes the reduced sprouting. We also proved that RA regulates glucose metabolism to affect endothelial cell angiogenesis.

Previous studies have shown that angiogenesis is genetically regulated; for instance, Notch signaling controls angiogenesis by inhibiting sprouting. Hence, blockade of Notch signaling induces vascular hyper-sprouting, while activation of the Notch pathway causes the opposite effect. Recent studies have shown that metabolism is also a crucial factor in angiogenesis. Glycolysis is the central node among all metabolic processes, and PFKFB3 is the key gene that induces angiogenesis in ECs. It has been suggested that PFKFB3 overexpression promotes angiogenesis but that silencing PFKFB3 causes the opposite effect, indicating that glycolysis regulates angiogenesis [4, 27]. Previous data demonstrated that shear stress-mediated repression of endothelial cell metabolism via KLF2 and PFKFB3 controls the endothelial cell phenotype [5]. Here, we found that RA decreases sprouting by upregulating FBP1, and loss-of-function studies with FBP1 knockdown and pharmacological inhibition highlights that FBP1, rather than other genes in glucose metabolism, regulates angiogenesis via RA stimulation. FBP1 has been reported to be a critical oncogene in some cancers. It has been indicated that loss of FBP1 induces glycolysis and results in increased glucose uptake, promoting tumorigenesis. These data are consistent with our results; we found that RA increases FBP1 expression and subsequently suppresses glycolysis and glucose uptake. In addition, our findings also suggested that by competing with PFKFB3 (as an activator of glycolysis), FBP1 functions as a negative regulator of glycolysis and can also regulate angiogenesis.

## Conclusions

RA not only reduces EC proliferation and migration but also impairs EC angiogenesis. More importantly, RA was found to upregulate the expression of FBP1 by directly binding to the FBP1 promoter via RARs, cause increased gluconeogenesis, and contribute to reduced sprouting in hESC-ECs. Prospectively, we provided a novel target for antiangiogenic therapy in angiogenesis-related pathological processes.

## Supplementary Information

**Additional file 1.** The supplemental information realted to the main text including Table S1–S4 and Figure S1–S6.

## Data Availability

The sequence data reported in this paper have been deposited in the Genome Sequence Archive (Genomics, Proteomics & Bioinformatics 2021) in National Genomics Data Center (Nucleic Acids Res 2022), China National Center for Bioinformation/Beijing Institute of Genomics, Chinese Academy of Sciences (GSA-Human: HRA002264) that are publicly accessible at https://ngdc.cncb.ac.cn/gsa-human.

## Abbreviations

RA: Retinoic acid
RAR: Retinoic acid receptor
hESCs: Human embryonic stem cells
ECs: Endothelial cells
hESC-ECs: Human embryonic stem cell-derived endothelial cells
FBP1: Fructose-1,6-bisphosphatase 1
