# Supplementary material for: Retinoic acid inhibits the angiogenesis of human embryonic stem cell-derived endothelial cells by activating FBP1-mediated gluconeogenesis
Source: Stem Cell Res Ther. 2022 Jun 7;13:239. doi: 10.1186/s13287-022-02908-x (PMC9171939; doi:10.1186/s13287-022-02908-x)
Supplement: Supplementary file 1 — Additional file 1. The supplemental information realted to the main text including Table S1–S4 and Figure S1–S6. [file 13287_2022_2908_MOESM1_ESM.docx]

**ADDITIONAL FILE 1**

**Retinoic acid inhibits the angiogenesis of** **human embryonic stem cell-derived endothelial cells by activating FBP1-mediated gluconeogenesis**

Zhuangzhuang Yang^1, #^, Miao Yu^1, #^, Xuechun Li^1, #^, Yuanyuan Tu^1, #^, Chunyan Wang^2, #^, Wei Lei^1^, Min Song^1^, Yong Wang^1^, Ying Huang^1^, Fengyue Ding^1^, Kaili Hao^1^, Xinglong Han^1^, Xuan Ni^1^, Lina Qu^2, *^, Zhenya Shen^1, *^, Shijun Hu^1, *^

^1^ Department of Cardiovascular Surgery of the First Affiliated Hospital & Institute for Cardiovascular Science, Collaborative Innovation Center of Hematology, State Key Laboratory of Radiation Medicine and Protection, Suzhou Medical College, Soochow University, Suzhou 215000, China; ^2^ State Key Laboratory of Space Medicine Fundamentals and Application, China Astronaut Research and Training Center, Beijing 100094, China

^#^ These authors contributed equally to this work

**ADDITIONAL FILE MATERIALS AND METHODS**

***Cell cultu******re.*** Human umbilical vein endothelial cells (HUVECs) were cultured in ECM (ScienCell, USA) supplemented with 50 ng/mL VEGF, 25 ng/mL bFGF and 2% FBS on 0.1% gelatin-coated dishes and passaged at 70-80% confluence with 0.1% trypsin. All the cells were maintained in a 37°C humidified incubator with 5% CO_2_.

***Tube formation assay in vitro.*** For *in vitro* tube formation, ECs were cultured on a solid layer of growth factor-reduced Matrigel in a 96-well plate (1×10^4^ cells per well) in ECM. Images were captured under an Olympus LX51 ﬂuorescence microscope (Olympus, Japan). The numbers of tubes were counted using the ImageJ software.

***CCK8 cell proliferation assay.*** 1×10^4^ cells were seeded on 96-well plate and cultured in 100 μL CCK8 assay medium consisting of 10 μL CCK8 solution and 90 μL culture medium. After incubation for 2 hours at 37°C, the cell culture medium was analyzed for 450 nm absorption using a Synergy H1 Hybrid Multi-Mode Microplate Reader (BioTek, USA).

***β-galactosidase staining assay.*** Cells in each group were seeded into a 12-well plate. After 24 hours of treatment, the culture medium was removed, and the cells were washed once with PBS. In accordance with the instructions of the senescence β-galactosidase Staining kit (Beyotime, China), SA-β-gal (1 ml) was added into cells for incubation overnight at 37 °C. Positive cells presented blue.

**
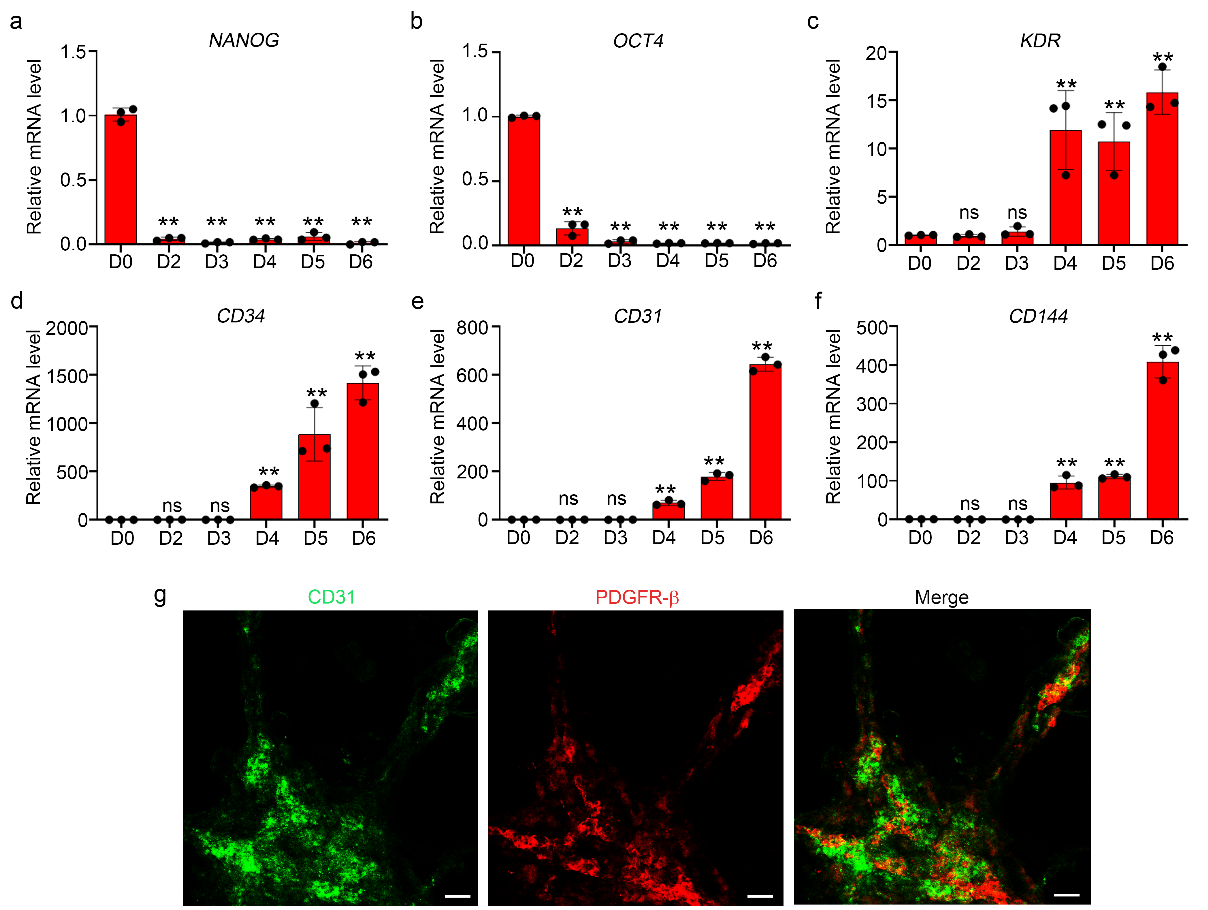
**

**Figure S1. Identification of endothelial cells differentiation from human embryonic stem cells**

(a-f) The qPCR analysis of the pluripotency markers *NANOG*, *OCT4* and endothelial markers *KDR*, *CD34*, *CD31* and *CD144* during endothelial differentiation from Day 0 to Day 6. (g) Representative photographs of immunocytochemistry of human CD31 (green) and PDGFR-β (red). Scale bars: 100 µm. All data are presented as mean ± SEM; One-way ANOVA; ***p* < 0.01 and ns, not significant.

**
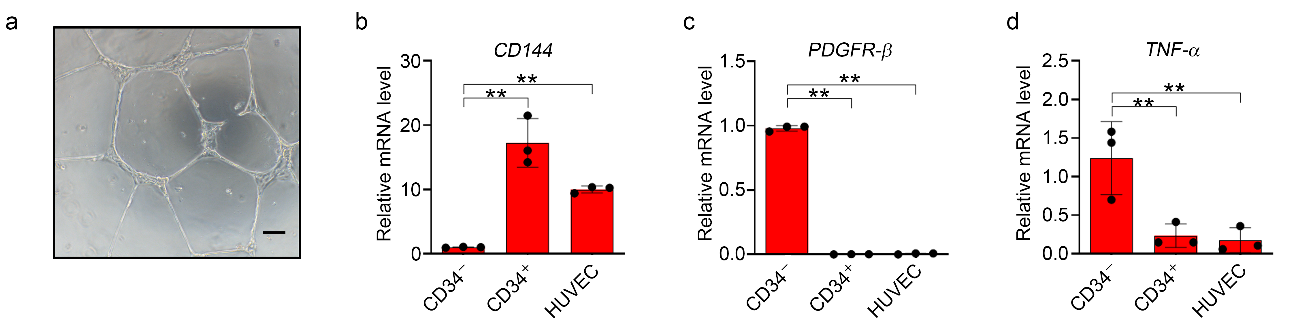
**

**Figure S2. Comparation of hESC-ECs and HUVECs**

(a) Representative photograph of cord-like structures on Matrigel formed by hESC-ECs. Scale bars, 100 μm. (b-d) The qPCR analysis showed profiles of transcripts related to endothelial and pericyte phenotypes among CD34^+^ ECs derived from ESCs, CD34^-^ cells and HUVECs. All data are presented as mean ± SEM; One-way ANOVA; ***p* < 0.01.


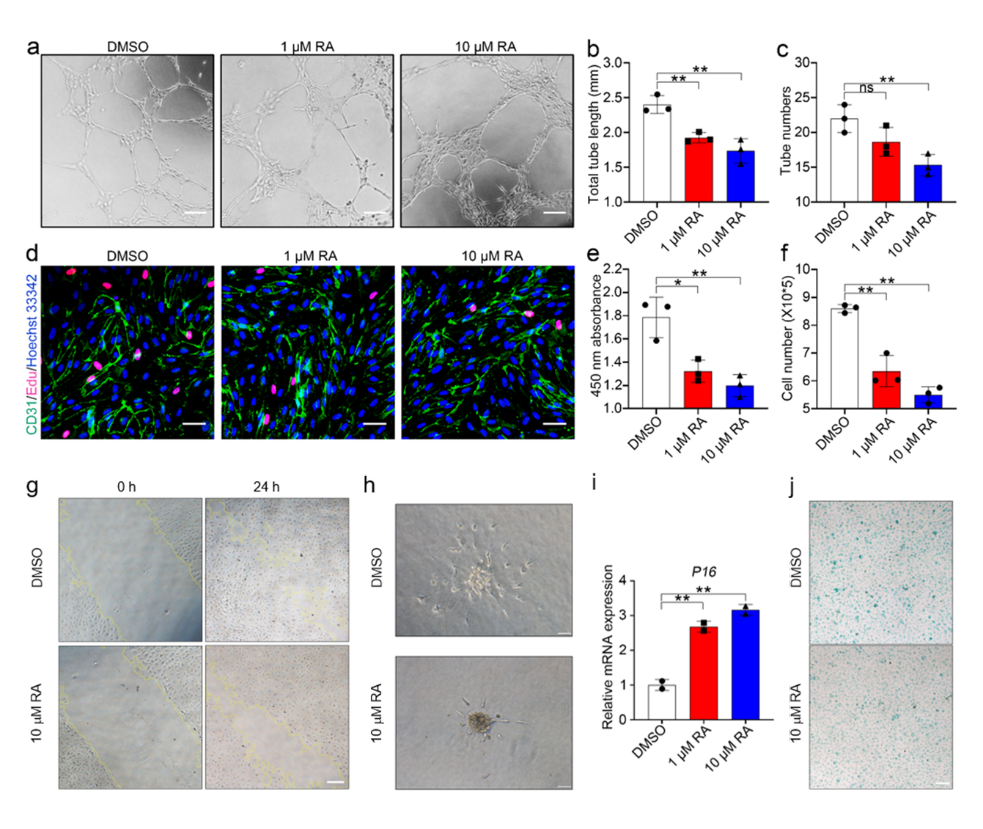


**Figure S3. RA inhibited endothelial tube formation and proliferation**

(a) Representative bright field photographs of tube formation by ECs treated with RA or not. Scale bars, 100 μm. Statistical analysis of tube length (b) and tube numbers (c) for panel a. (d) Staining for EdU (red), CD31 (green) and Hoechst 33342 (blue) in ECs after indicated treatment. Scale bars, 50 μm. CCK8 analysis (e) and cell number counting (f) to indicate the ability of proliferation after indicated treatment. (g) Representative photographs of wound healing for HUVEC. Scale bars, 100 μM. (h) Representative photographs of endothelial spheroids for HUVEC. Scale bar, 50 μm. (i) The qPCR analysis of the expression of the CDK inhibitor *P16* in different groups. (j) Representative photographs of cell senescence. Scale bar, 200 μm. All data are presented as mean ± SEM; One-way ANOVA; **p* < 0.05, ***p* < 0.01 and ns, not significant.

**
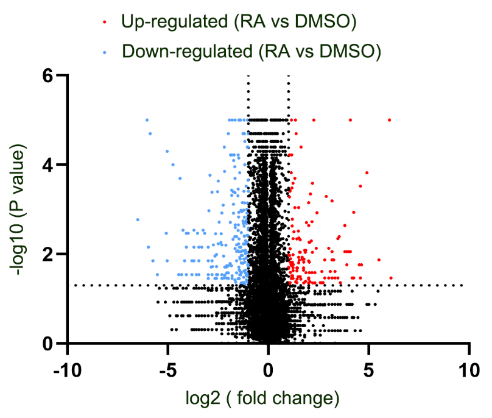
**

**Figure S4. A volcano diagram for RNA-seq data.**

Volcano diagram of RNA-seq data (p < 0.05, fold change ≥ 2) in RA-treated hESC-ECs versus control hESC-ECs. Red dots represent up-regulated genes blue dots represents down-regulated genes. Blank ones are not significant differentially expressed genes

**
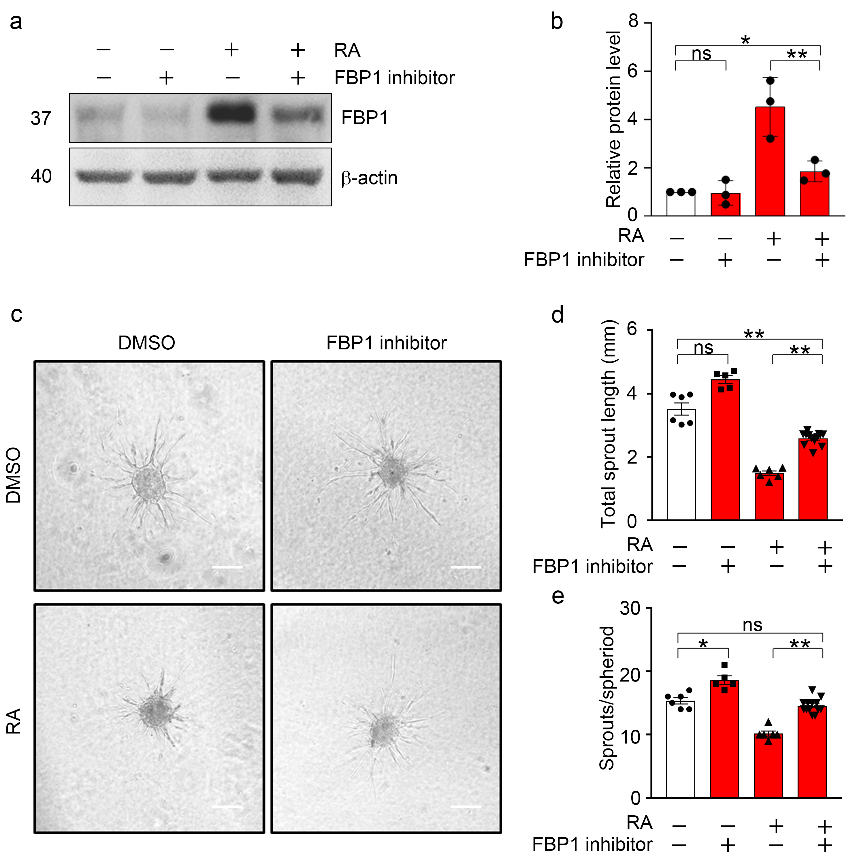
**

**Figure S5. FBP1 inhibitor restored endothelial sprouting**

(a) The expression of FBP1 in ECs after indicated treatment. (b) Quantification of the FBP1 protein level in panel a. (c) Representative bright field photographs of EC spheroids after indicated treatment. Scale bars, 50 μm. Statistical analysis of total sprout length (d) and sprout number of each spheroid (e) for panel c. All data are presented as mean ± SEM; One-way ANOVA; **p* < 0.05, ***p* < 0.01 and ns, not significant.

**
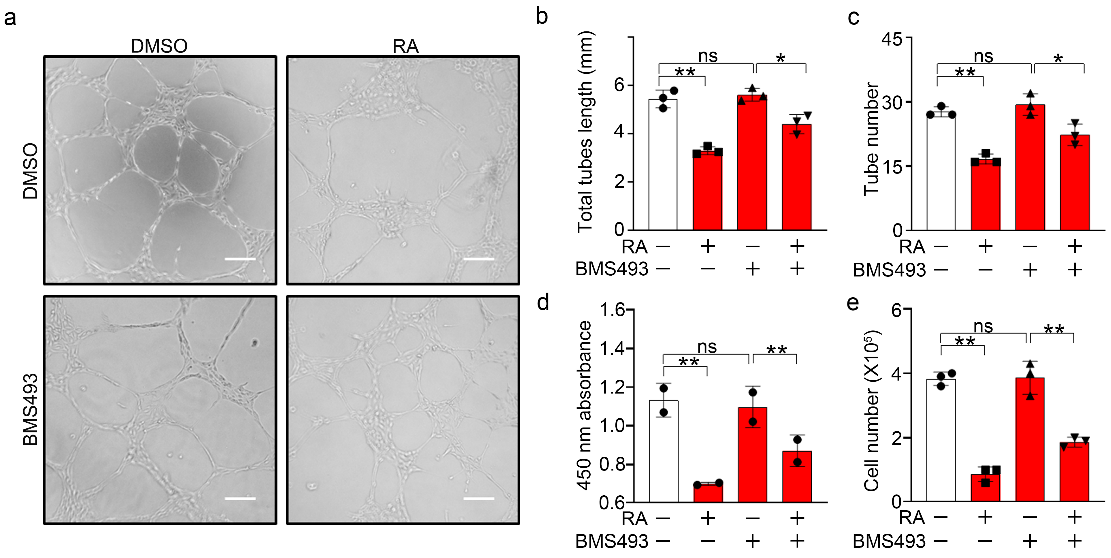
**

**Figure S6. RA inhibitor BMS493 restored endothelial tube formation and proliferation**

(a) Representative bright field photographs of tube formation. Scale bars, 100 μm. Statistical analysis of total tube length (b) and tube numbers (c) for panel a. CCK8 analysis of the cell number (d) and cell number counting (e). All data are presented as mean ± SEM; One-way ANOVA; **p* < 0.05, ***p* < 0.01 and ns, not significant.**Table S1. Antibodies for immunofluorescence (IF), flow cytometry (FCM) and western blot (WB).**

| **Antibody** | **Company** | **Catalog Number** | **IF** | **FCM** | **WB** |
| --- | --- | --- | --- | --- | --- |
| CD31 | Abcam | Ab28364 | 1:200 |  |  |
| PDGFR-β | Proteintech | 13449-1-AP | 1:200 |  |  |
| CD34-APC | Invitrogen | 555824 |  | 1:200 |  |
| CD144-PE | Invitrogen | 12-1449-82 |  | 1:200 |  |
| β-actin  FBP1 | Proteintech  Proteintech | 66009-1-LG  12842-1-AP |  |  | 1:5000  1:5000 |
| Alexa Fluor^®^ 488 AffiniPure Donkey Anti-Mouse IgG (H+L) | Jackson ImmuoResearch | 715-545-151 | 1:1000 |  |  |
| Alexa Fluor^®^ 594 AffiniPure Donkey Anti-Rabbit IgG (H+L) | Jackson ImmuoResearch | 711-585-152 | 1:1000 |  |  |

**Table S2. T****he primer list for the qPCR.**

| **Genes** | **Forward (5’→3’)** | **Reverse (5’→3’)** |
| --- | --- | --- |
| *NANOG* | ATAGATAAGTAGATCTAATAC | AAACGGTAAGAAATCAATTAA |
| *OCT4* | GTGGAGGAAGCTGACAACAA | ATTCTCCAGGTTGCCTCTCA |
| *KDR* | CGGCTCTTTCGCTTACTGTT | TCCTGTATGGAGGAGGAGGA |
| *CD34* | CAAGCCACCAGAGCTATTCC | TAGCCAGTGATGCCCAAGAC |
| *CD31* | TCTATGACCTCGCCCTCCACAAA | GAACGGTGTCTTCAGGTTGGTATTTCA |
| *CD144* | CTGGCCATGGACCCTGATG | CGGAAGAACTGGCCCTTGT |
| *PDGFR-β* | TGGCAGAAGAAGCCACGTT | GGCCGTCAGAGCTCACAGA |
| *TNF-α* | AGCCCCCAGTCTGTATCCTT | CTCCCTTTGCAGAACTCAGG |
| *18S rRNA* | GTAACCCGTTGAACCCCATT | CCATCCAATCGGTAGTAGCG |
| *P16* | AGCTGTCGACTTCATGACAAG | GAGCTTTGGTTCTGCCATTTG |
| *FBP1* | AGTGCCTACTGCCCTCTCTTG | GCCATGCTTGAACCGGGTA |
| *HK* | TGCCACCAGACTAAACTAGACG | CCCGTGCCCACAATGAGAC |
| *G6Pase* | TTCCGTGCCCCTGATAAAGC | AGTATACACCTGCTGTGCCCAT |
| *PK* | ATGTCGAAGCCCCATAGTGAA | TGGGTGGTGAATCAATGTCCA |
| *PFK1* | GAGCACCATGCAGCCAAAAC | GCAGCATTCATACCTTGGGC |
| *HK* | GTGAATCGGAGAGGTCCCAC | CAAGCAGATGCGAGGCAATC |
| *PC* | GCGACGGCGAGGAGATAGT | GCTTCATCTGCTTTCTGCCG |
| *PK* | CCTGATAGCTCGTGAGGCTG | TTGAGGCTCGCACAAGTTCT |
| *PEPCK* | TCTTCTAGGACTGCCAGGAGG | CTGGTGGAGTTGGGACATGA |
| *PFKFB3* | GATGCCCTTCAGGAAAGCCT | GAACACTTTTGTGGGGACGC |

**Table S3. The primer list for ChIP-PCR.**

| **Target Region** | **Primer Sequence (5’→3’)** | **Length (bp)** |
| --- | --- | --- |
| *RARE1* | Forward: GATTGCAGCTGAGGAACCATG  Reverse: TACATCCATGTTTGGGAGGCC | 122 |
| *RARE2* | Forward: AAATGCAATTTGTTGCAAGCA  Reverse: ATCAATTTCCTGCTGGGAAAA | 279 |
| *RARE3* | Forward: AGGATGGGTCCTCCTCTGAAG  Reverse: CTTCTGGTCCCAGGCTGACAT | 107 |
| *GAPDH* | Forward: GAAGGTGAAGGTCGGAGT  Reverse: GATGGCAACAATATCCACTT | 94 |

**Table S4. The oligomer list for FBP1-selective RNA interference (siRNA)**

| **Genes** | **Sense (5’→3’)** | **Antisense (5’→3’)** |
| --- | --- | --- |
| FBP1-Homo-662 | GGGUAAAUAUGUGGUCUGUTT | ACAGACCACAUAUUUACCCTT |
| FBP1-Homo-741 | GGCAUCUAUAGAAAGAAAUTT | AUUUCUUUCUAUAGAUGCCTT |
